# Coordinated Expression of ADC Targets *ERBB2*, *TACSTD2*, and *NECTIN4* in Bladder Cancer: A Multi-Scale Transcriptomic and Proteomic Landscape Analysis

**DOI:** 10.3390/cimb48080825

**Published:** 2026-08-13

**Authors:** Sanhe Liu, Liqun Duan, Shaozhong Wei

**Affiliations:** 1 College of Biomedicine and Health, Huazhong Agricultural University, Wuhan 430070, China; sanheliu@whu.edu.cn; 2 Department of Urology, Hubei Cancer Hospital, Tongji Medical College, Huazhong University of Science and Technology, Wuhan 430079, China; 3 Division of Infection and Immunity, Systems Immunity Research Institute, School of Medicine, Cardiff University, Cardiff CF14 4XN, UK; qunqunduan1314@gmail.com; 4 Department of Radiation Oncology, Hubei Cancer Hospital, Tongji Medical College, Huazhong University of Science and Technology, Wuhan 430079, China

**Keywords:** bladder cancer, antibody–drug conjugate, *ERBB2*, *TACSTD2*, *NECTIN4*, spatial transcriptomics, multiplex immunofluorescence, co-expression

## Abstract

Antibody–drug conjugates (ADCs) targeting HER2 (encoded by *ERBB2*), Trop2 (*TACSTD2*), and Nectin-4 (*NECTIN4*) have demonstrated clinical activity in bladder cancer. However, the co-expression relationships among these three targets at both the transcriptomic and protein levels, and their spatial organization within the tumor microenvironment, remain incompletely characterized. To address this, we performed a multi-platform, multi-resolution analysis integrating ten bulk RNA-seq datasets (*n* = 1628 samples), six spatial transcriptomics datasets (10x Visium and Visium HD, *n* = 31 specimens), and multiplex immunofluorescence (mIF) staining on three paired tumor–adjacent normal bladder specimens. Pairwise Pearson correlation analyses were conducted for *ERBB2*, *TACSTD2*, and *NECTIN4* across bulk transcriptomes and spatial compartments (tumor core, interface, and stroma). High-resolution co-expression hotspot mapping was performed using Visium HD data. Preliminary protein-level evidence was obtained using mIF, with quantitative three-dimensional surface plot reconstructions illustrating the spatial distribution of marker co-expression. Given the limited number of specimens (three paired tumor–normal samples), these findings should be regarded as exploratory and illustrative rather than definitive validation. Moderate-to-strong positive correlations among the three genes were consistently observed across all ten bulk datasets, with the strongest association between *TACSTD2* and *NECTIN4* (Pearson r up to 0.969). Spatial transcriptomic analyses recapitulated these positive correlations across all histopathological compartments, with the tumor core generally exhibiting the highest coefficients. No consistent spatial gradient was observed, indicating preservation of co-expression across tissue niches. Visium HD analysis confirmed positive pairwise correlations and identified discrete co-expression hotspots. mIF revealed extensive Trop2–Nectin-4 protein colocalization across all tumors, whereas HER2 showed more restricted and heterogeneous expression. Triple-positive subpopulations were identified but were limited. Strikingly, all three markers exhibited pronounced tumor-specific upregulation relative to paired adjacent normal tissues, with surface plot analysis showing markedly elevated cumulative fluorescence intensities in tumors (Z-values approaching 240 arbitrary units) compared with a flattened landscape in normal tissues (Z-values < 100–120 arbitrary units). These findings provide a multi-scale description of ADC-target co-expression in bladder cancer and generate hypotheses for biomarker-driven patient stratification, which require validation in larger, independent cohorts.

## 1. Background

Bladder cancer constitutes a major worldwide oncological burden, accounting for roughly 573,000 incident diagnoses and 213,000 fatalities each year [1]. Even with progress in surgical techniques and systemic regimens—including platinum-containing chemotherapy and immune checkpoint blockade—outcomes for individuals presenting with advanced or metastatic disease remain unfavorable. Five-year survival approximates 15% for muscle-invasive bladder cancer (MIBC) and falls below 10% once distant spread has occurred [2,3]. Over the past decade, antibody–drug conjugates (ADCs) have gained traction as a compelling treatment paradigm, harnessing tumor-restricted cell-surface molecules to deliver cytotoxic warheads selectively to malignant cells [4]. Three ADC antigens under particularly intense investigation in bladder cancer are human epidermal growth factor receptor 2 (HER2, encoded by *ERBB2*), trophoblast cell-surface antigen 2 (Trop2, encoded by *TACSTD2*), and nectin cell adhesion molecule 4 (Nectin-4, encoded by *NECTIN4*).

HER2 is an established oncogenic driver in breast and gastric carcinomas [5], and its relevance to bladder cancer has drawn growing scrutiny [6,7]. Anti-HER2 ADCs such as trastuzumab deruxtecan (T-DXd) and disitamab vedotin (RC48) have produced clinical signals in HER2-overexpressing as well as HER2-low bladder tumors [8,9,10,11]. Trop2, a transmembrane glycoprotein involved in intracellular calcium signaling, is broadly upregulated across epithelial cancers, including urothelial neoplasms [12]. Sacituzumab govitecan (SG)—an anti-Trop2 ADC bearing the topoisomerase I inhibitor SN-38—has yielded encouraging efficacy data in metastatic urothelial carcinoma [13,14,15,16]. Nectin-4 belongs to the nectin family of adhesion molecules and displays overexpression in bladder cancer; it serves as the molecular target for enfortumab vedotin (EV), an ADC armed with the microtubule-disrupting agent monomethyl auristatin E (MMAE). EV has already secured regulatory approval for locally advanced or metastatic urothelial carcinoma and is being explored in earlier lines of therapy [17,18,19,20,21,22].

Although each of these three molecules has been profiled individually, how their expression correlates—at both the mRNA and protein tiers—in bladder cancer has not been thoroughly delineated. Elucidating whether *ERBB2*, *TACSTD2*, and *NECTIN4* exhibit concerted expression patterns and how that behavior is organized across the tumor microenvironment’s spatial architecture has direct implications for therapeutic decision-making. The degree and spatial pattern of co-expression may help determine whether combinatorial or sequential ADC regimens are warranted, whether patient stratification should incorporate multi-target expression signatures, and whether the therapeutic index of these agents is buttressed by selective overexpression in tumor relative to normal tissue.

Recent progress in spatial transcriptomics permits profiling of gene activity within intact tissue architecture, conserving positional cues that conventional bulk sequencing discards. Platforms such as 10x Visium and Visium HD offer resolution sufficient to examine how expression relationships shift across distinct histopathological zones—the tumor core, the tumor–stroma boundary, and the stromal compartment—and to pinpoint fine-grained co-expression hotspots [23]. In parallel, mIF enables concurrent visualization of several protein targets, delivering orthogonal corroboration at the translational level.

Here, we undertook a systematic multi-platform, multi-resolution characterization of *ERBB2*, *TACSTD2*, and *NECTIN4* expression and co-expression in bladder cancer. Our approach spanned bulk RNA-seq, spatial transcriptomics (10x Visium and Visium HD), and mIF, collectively covering 1628 bulk transcriptomes and 31 spatial specimens with matched normal tissue comparisons, providing a multi-scale, descriptive characterization of the co-expression landscape of ADC targets and generating testable biological and clinical hypotheses.

## 2. Materials and Methods

### 2.1. Data Preparation and Processing

Ten bulk RNA-seq collections were sourced from publicly accessible repositories. The TCGA-BLCA cohort was retrieved through the Genomic Data Commons portal. The IMvigor210 dataset, originating from the IMvigor210 trial, was acquired via the IMvigor210CoreBiologies R package (v2.0.0) and restricted to bladder cancer specimens. E-MTAB-1803 was downloaded from ArrayExpress (EMBL-EBI, accession E-MTAB-1803). The remaining seven datasets—GSE31684, GSE133624, GSE216037, GSE236932 [24], GSE248167, GSE279390, and GSE328930—were obtained from the Gene Expression Omnibus (GEO). All expression values were transformed to log2(TPM + 1) before correlation computations. Spatial transcriptomic resources—Visium datasets GSE285715, GSE246011, and GSE319536, plus the Visium HD dataset GSE238145—were retrieved from GEO. The Visium dataset E-GEAD-1186 was sourced from the Genomic Expression Archive (GEA) at the DNA Data Bank of Japan (DDBJ), accession E-GEAD-1186.

All ten bulk RNA-seq datasets were verified to represent independent patient cohorts with no sample overlap. Specifically, TCGA-BLCA (*n* = 408) contains unique patients from The Cancer Genome Atlas; IMvigor210 (*n* = 195) originates from a single phase II clinical trial; E-MTAB-1803 (*n* = 73) represents an independent European cohort; and the seven GEO datasets (GSE31684, *n* = 93; GSE133624, *n* = 65; GSE216037, *n* = 52; GSE236932, *n* = 63; GSE248167, *n* = 51; GSE279390, *n* = 562; GSE328930, *n* = 66) were generated by distinct research groups using non-overlapping patient collections. The total of 1628 samples, therefore, represents 1628 unique patient tumor specimens. A detailed summary of each cohort is provided in Table 1 (cohort summary table).

Integration of multiple independent cohorts was undertaken to assess the reproducibility and generalizability of co-expression relationships across heterogeneous clinical contexts. Rather than assuming batch homogeneity, we explicitly treated each dataset as an independent replication opportunity: the consistency of correlation patterns across cohorts—despite differences in patient demographics, disease stage distribution, sequencing platforms, and laboratory protocols—serves as the primary strength of this integrative approach, as concordant findings across heterogeneous datasets are substantially less likely to reflect technical artifacts or cohort-specific bias. Importantly, each dataset was analyzed independently; no batch correction or data pooling was performed prior to correlation analysis.

### 2.2. Sample Collection and Processing

Fresh surgical specimens of tumor and matched peritumoral tissue were procured from three subjects with histologically verified bladder cancer, all collected postoperatively within a one-year window. Tissues were preserved at −80 °C. Clinical and pathological characteristics are cataloged in Table 2. The study received approval from the Institutional Review Board of Hubei Cancer Hospital (Approval No. LLHBCH2026YN-091), and written informed consent was obtained from every participant.

### 2.3. Identification of Spatial Tumor–Immune Interfaces

Spatial tumor–immune interfaces in the 10x Visium datasets were delineated by evaluating the colocalization of malignant and immune cell proportions across tissue spots through the SpaCET R package (https://github.com/data2intelligence/SpaCET, 1 August 2026) [25].

### 2.4. Bulk RNA-Seq Correlation Analysis and Visualization

All computational analyses and graphics were produced with Python 3.10. Core dependencies included scanpy (v1.11.5), pandas (v2.3.3), numpy (v1.26.4), scipy (v1.15.3), seaborn (v0.13.2), matplotlib (v3.10.8), anndata (v0.11.4), and pathlib (v3.4). Correlation matrices were displayed as heatmaps employing seaborn.heatmap, with hierarchical clustering carried out through seaborn.clustermap under Euclidean distance and average linkage (UPGMA).

### 2.5. Spatial Transcriptomic (10x Visium) Correlation Analysis

Data from the 10x Visium platform were handled with the standard scanpy pipeline (anndata v0.11.4). For each tissue section, spots were classified into three histological zones: tumor core, interface (tumor–stroma border), and stroma. Expression values were normalized as log2(TPM + 1). Pairwise Pearson coefficients for *ERBB2*, *TACSTD2*, and *NECTIN4* were calculated across all spots belonging to each zone. Two-sided *p*-values were derived from Pearson tests and annotated as *** *p* < 0.001, ** *p* < 0.01, * *p* < 0.05, blank or ns (not significant). Heatmap visualization and hierarchical clustering were implemented as described above. Spot counts per region, correlation coefficients, and associated *p*-values were collated.

### 2.6. Spatial Transcriptomic (10x Visium HD) Correlation Analysis

These analyses were conducted in R v4.3.1 with the following packages: Seurat (v4.3.0), ggplot2 (v4.0.1), patchwork (v1.3.2.9000), dplyr (v1.2.1), and corrplot (v0.95). Seurat object construction and quality filtering adhered to standard procedures. Normalization was accomplished with SCTransform (v0.4.3). Principal component analysis (PCA) was run on scaled residuals (RunPCA), and the first 30 PCs were retained for downstream steps. Neighbor graph assembly and clustering employed FindNeighbors and FindClusters (resolution = 0.5). Uniform Manifold Approximation and Projection (UMAP) was applied for non-linear dimensionality reduction (RunUMAP, dims = 1:30).

Spatial coordinates were pulled from Seurat metadata and merged with cluster assignments to produce spatial scatter diagrams via ggplot2. Normalized expression of *ERBB2*, *TACSTD2*, and *NECTIN4* was extracted (GetAssayData, slot = “data”). Pairwise Pearson coefficients were determined across the full set of spots.

### 2.7. Multiplex Immunofluorescence (mIF) Staining

mIF staining was carried out on formalin-fixed paraffin-embedded (FFPE) sections to permit simultaneous detection of several proteins within a single tissue slice. The antibody panel comprised HER2 (clone EPR19547-46, 1:250, Abcam, Cambridge, UK), Trop2 (clone EPR20043, 1:500, Abcam), and Nectin-4 (clone EPR15613-68, 1:500, Abcam), with DAPI serving as nuclear counterstain.

Briefly, FFPE sections underwent deparaffinization in xylene and rehydration across a descending ethanol series (100%, 95%, 75%). Antigen retrieval was performed in a pressure cooker for 2 min using citrate buffer (pH 6.0) or EDTA buffer (pH 9.0). After equilibration to ambient temperature, sections were rinsed three times with Tris-buffered saline (TBS), 5 min per wash. Endogenous peroxidase was quenched with 3% hydrogen peroxide, after which 10% normal donkey serum was applied for 30 min at room temperature to limit non-specific binding. Sections were then exposed to primary antibodies overnight at 4 °C. Following TBS washes, HRP-conjugated secondary antibodies (1:400) were applied for 1 h at room temperature (or 30 min at 37 °C) under light-protected conditions. Tyramide signal amplification (TSA) utilized Opal fluorophores (Akoya Biosciences, Marlborough, MA, USA). Between successive staining cycles, antibodies were stripped by microwave heating in citrate buffer, preserving the covalently attached TSA fluorophores. Nuclei were counterstained with 4′,6-diamidino-2-phenylindole (DAPI, 1:500) for 10 min at room temperature in the dark. Sections were coverslipped with anti-fade mounting medium and imaged on a multispectral platform (Vectra Polaris, Akoya Biosciences). Spectral unmixing was employed to resolve individual fluorophore channels and subtract autofluorescence.

To generate quantitative visual renderings of spatial distribution and relative fluorescence across the combined markers, three-dimensional surface plot reconstructions were produced from the merged mIF images in ImageJ (National Institutes of Health, Bethesda, MD, USA) (https://imagej.net/ij/download.html, 1 August 2026).

## 3. Results

### 3.1. Expression of ERBB2, TACSTD2, and NECTIN4 Displays Moderate-to-Strong Balanced Correlations Across Multiple Bladder Cancer Bulk Transcriptomic Cohorts

**Figure 1:**
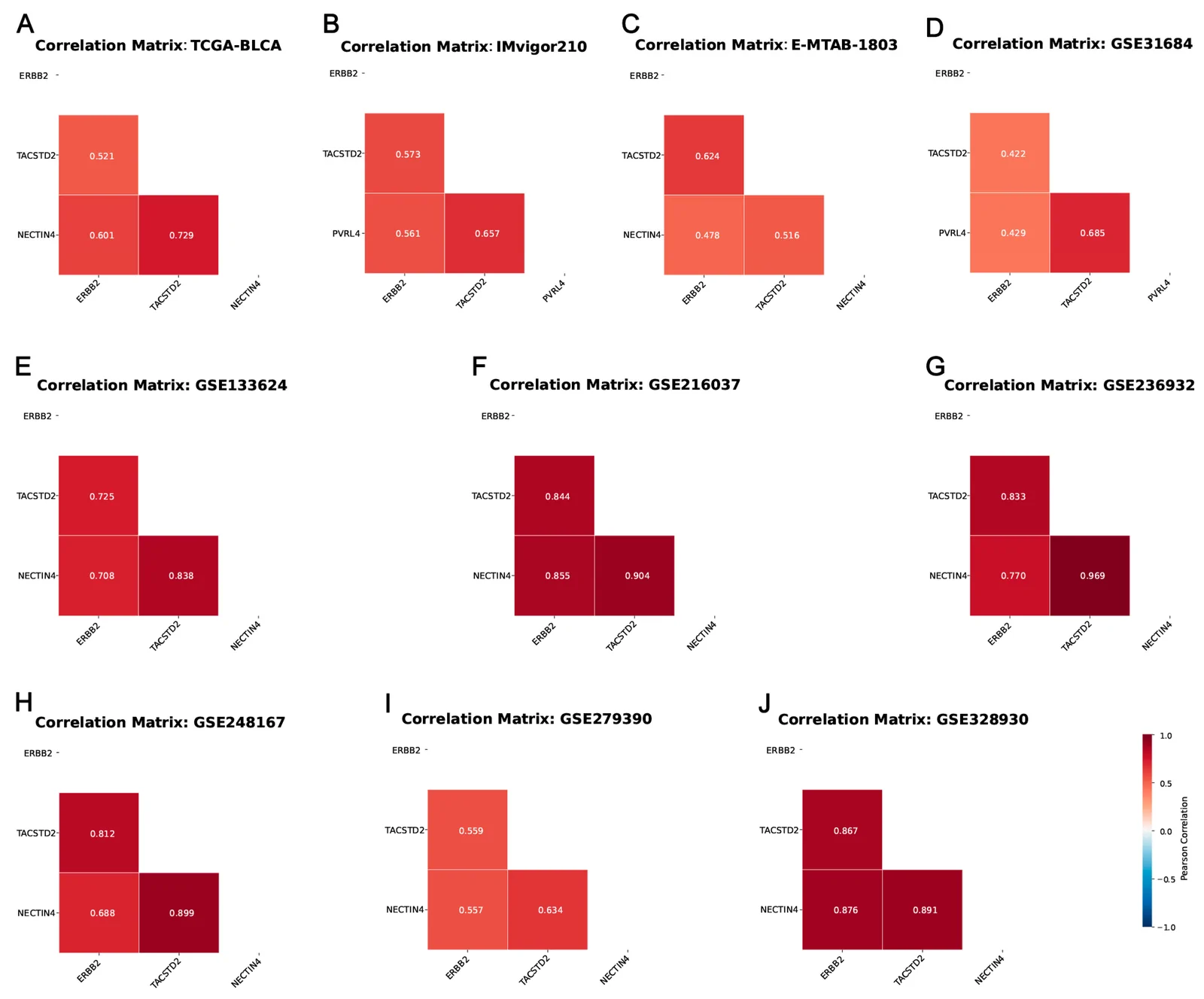
Correlation heatmaps of *ERBB2*, *TACSTD2*, and *NECTIN4* (*PVRL4*) expression (log2[TPM + 1]) across multiple bulk RNA-seq datasets in bladder cancer. (**A**) TCGA-BLCA *(n* = 408); (**B**) IMvigor210 (*n* = 195); (**C**) E-MTAB-1803 (*n* = 73); (**D**) GSE31684 (*n* = 93); (**E**) GSE133624 (*n* = 65); (**F**) GSE216037 (*n* = 52); (**G**) GSE236932 (*n* = 63); (**H**) GSE248167 (*n* = 51); (**I**) GSE279390 (*n* = 562); (**J**) GSE328930 (*n* = 66). (TPM = transcripts per million).

To assess the inter-relationships among *ERBB2*, *TACSTD2*, and *NECTIN4* (also designated *PVRL4*) at the transcript level in bladder cancer, we carried out pairwise correlation analyses on ten independent bulk RNA-seq collections encompassing 1628 specimens (Figure 1). Log-normalized expression (log2[TPM + 1]) for each gene was examined through pairwise correlation matrices.

Across datasets, moderate-to-strong positive associations were consistently detected. The tightest coupling was observed between *NECTIN4* and *TACSTD2*, with Pearson coefficients climbing as high as 0.969 in GSE236932 and 0.904 in GSE216037. The *ERBB2*–*TACSTD2* pair showed greater fluctuation, spanning from moderate (e.g., 0.422 in GSE31684) to comparatively strong (e.g., 0.867 in GSE328930). Likewise, *ERBB2*–*NECTIN4* correlations ranged from 0.429 in GSE31684 to 0.876 in GSE328930. Within each dataset, the three gene pairs maintained balanced correlation magnitudes without pronounced disparities.

Strikingly, no pair of genes registered significant negative correlations in any dataset, arguing against inverse regulatory relationships at the bulk transcriptomic scale. The overall correlation structure proved robust regardless of data provenance, cohort size, or clinical makeup (e.g., muscle-invasive versus non-muscle-invasive disease), underscoring the dependability of these co-expression observations.

Collectively, these data point to a coordinated transcriptional program linking the three genes in bladder cancer, most prominently between *TACSTD2* and *NECTIN4*. This provides a transcriptomic foundation for probing the functional interplay and therapeutic ramifications of this gene triad in urothelial carcinoma.

It is worth noting that Table 1 explicitly states the dataset accession numbers and the number of patients and confirms that each cohort is non-overlapping (same below).

### 3.2. Spatial Co-Expression of ERBB2, TACSTD2, and NECTIN4 Across Tumor Compartments in Bladder Cancer and Matched Normal Tissue

**Figure 2:**
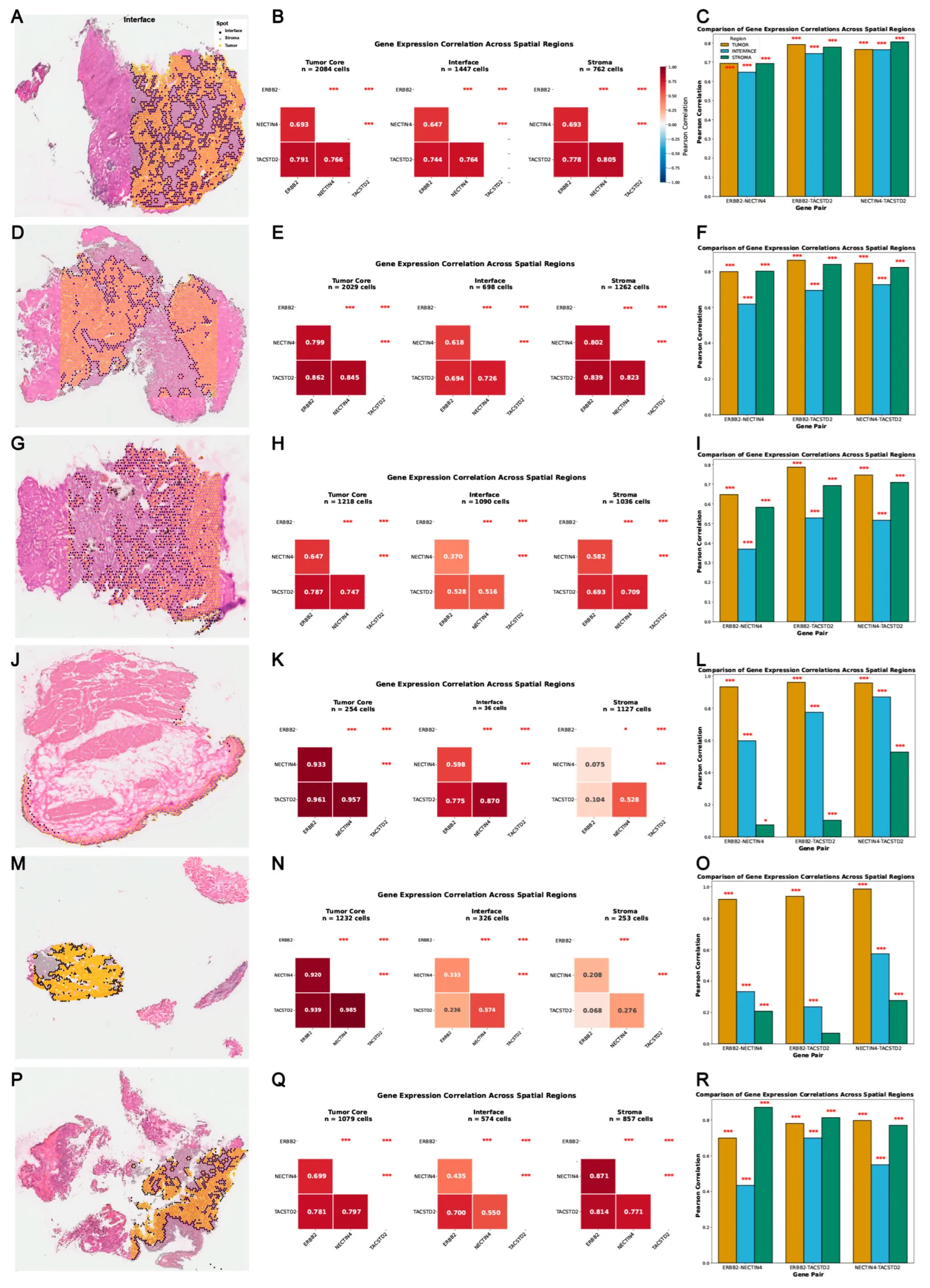
Correlations of *ERBB2*, *TACSTD2*, and *NECTIN4* expression across tumor core, interface, and stromal regions in the 10x Visium dataset GSE285715 (bladder cancer). The figure includes six samples: primary tumor tissues T1 (GSM8707234), T2 (GSM8707235), and T3 (GSM8707236); and adjacent normal tissues N1 (GSM8707237), N2 (GSM8707238), and N3 (GSM8707239). For each sample, panels (**A**,**D**,**G**,**J**,**M**,**P**) show spatial feature plots of gene expression distributions; panels (**B**,**E**,**H**,**K**,**N**,**Q**) display the corresponding correlation heatmaps; and panels (**C**,**F**,**I**,**L**,**O**,**R**) present bar plots of expression levels across the three tissue compartments. Two-sided *p*-values (Pearson correlation test) are denoted by asterisks: * *p* < 0.05, and *** *p* < 0.001; no asterisk, not significant.

To determine whether the bulk-level co-expression relationships persist within discrete spatial microenvironments of the tumor, we interrogated the 10x Visium spatial transcriptomic dataset GSE285715. This dataset contains three primary bladder tumor specimens (T1–T3) and three matched normal counterparts (N1–N3) (Figure 2). Spot-resolved gene expression was projected onto histological images partitioned into three zones—tumor core, interface, and stroma (spatial feature plots). Pairwise correlations were then determined (correlation heatmaps) and quantified across the three histologically delineated compartments (bar plots) (Figure 2).

Overall, positive gene–gene correlations were recapitulated at the spatial level across all specimens and tissue compartments. The *ERBB2*–*TACSTD2* pair, in particular, maintained strong associations throughout the tumor core, interface, and stroma in both the tumor and the adjacent specimen N3, with most Pearson coefficients approaching or surpassing 0.8. Correlations that involved *NECTIN4*—both with *TACSTD2* and with *ERBB2*—showed greater compartment-to-compartment and sample-to-sample variability while staying predominantly in the positive range.

In select specimens, modest regional differences in correlation magnitude were discernible. For example, within tumor samples, coefficients tended to be marginally higher in the core than in the stromal zone for certain gene pairs, with the interface registering the lowest values. Among adjacent samples, the pattern was more heterogeneous, although the tumor core regions still showed the highest coefficients—in some cases exceeding those measured in the tumor specimens themselves. These trends imply that the local microenvironment may fine-tune these co-expression relationships. Nonetheless, the absence of a uniform directional gradient across all six samples indicates that the overarching co-expression architecture is largely conserved across spatial niches.

These spatial transcriptomic analyses confirm that the coordinated expression of *ERBB2*, *TACSTD2*, and *NECTIN4* does not simply reflect a bulk-averaging artifact but is preserved at the tissue microenvironment scale, lending support to the relevance of all three targets for spatially guided therapeutic strategies in bladder cancer.

### 3.3. Spatial Co-Expression of ERBB2, TACSTD2, and NECTIN4 in Non-Muscle-Invasive Bladder Cancer

**Figure 3:**
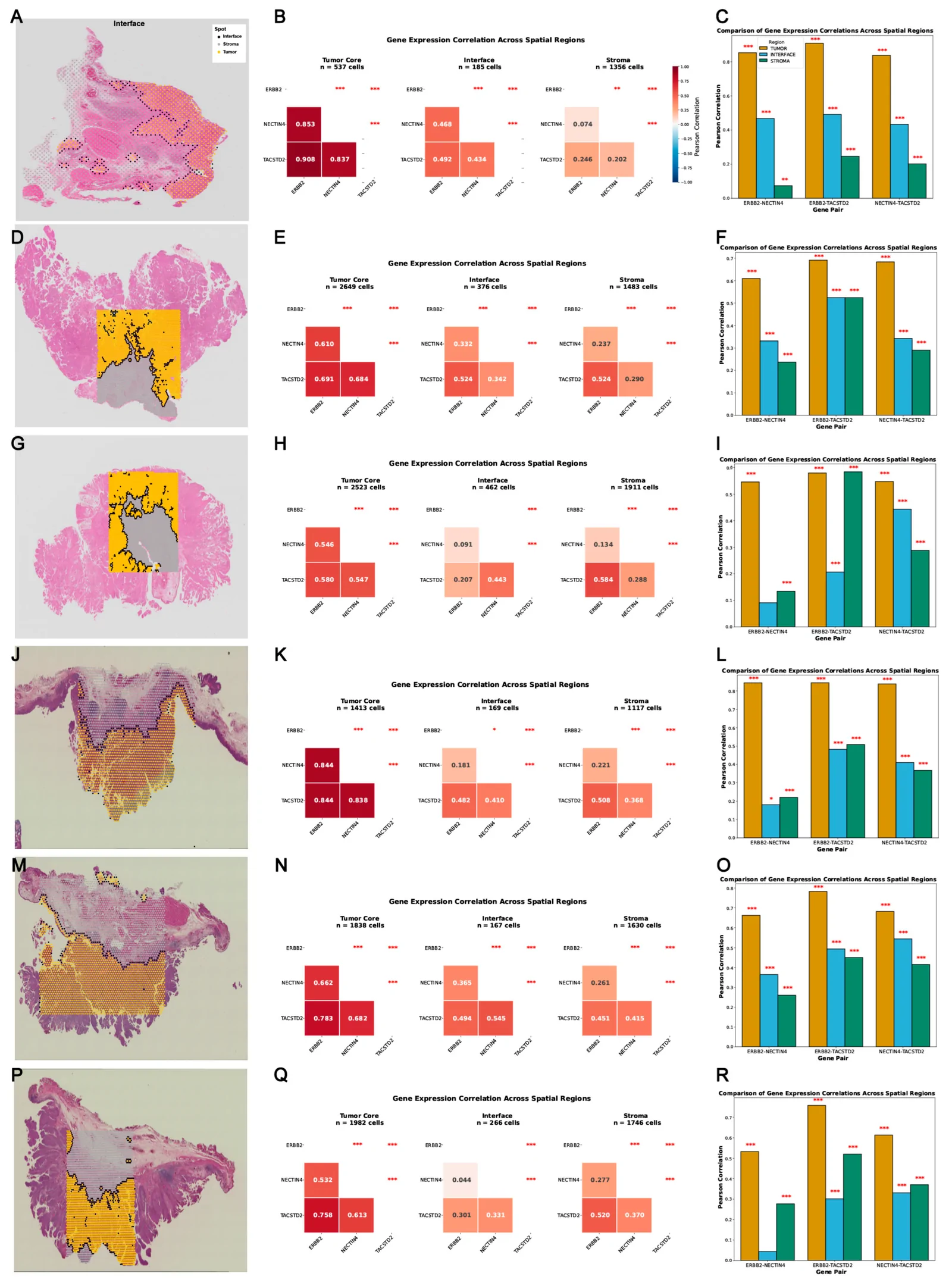
Correlations of *ERBB2*, *TACSTD2*, and *NECTIN4* expression across tumor core, interface, and stromal regions in the 10x Visium dataset E-GEAD-1186. The figure includes six NMIBC samples (samples 1–4, 6, and 7). For each sample, panels (**A**,**D**,**G**,**J**,**M**,**P**) show spatial feature plots of gene expression distributions; panels (**B**,**E**,**H**,**K**,**N**,**Q**) display the corresponding correlation heatmaps; and panels (**C**,**F**,**I**,**L**,**O**,**R)** present bar plots of expression levels across the three tissue compartments. Two-sided *p*-values (Pearson correlation test) are denoted by asterisks: * *p* < 0.05, ** *p* < 0.01, and *** *p* < 0.001; no asterisk, not significant.

**Figure 4:**
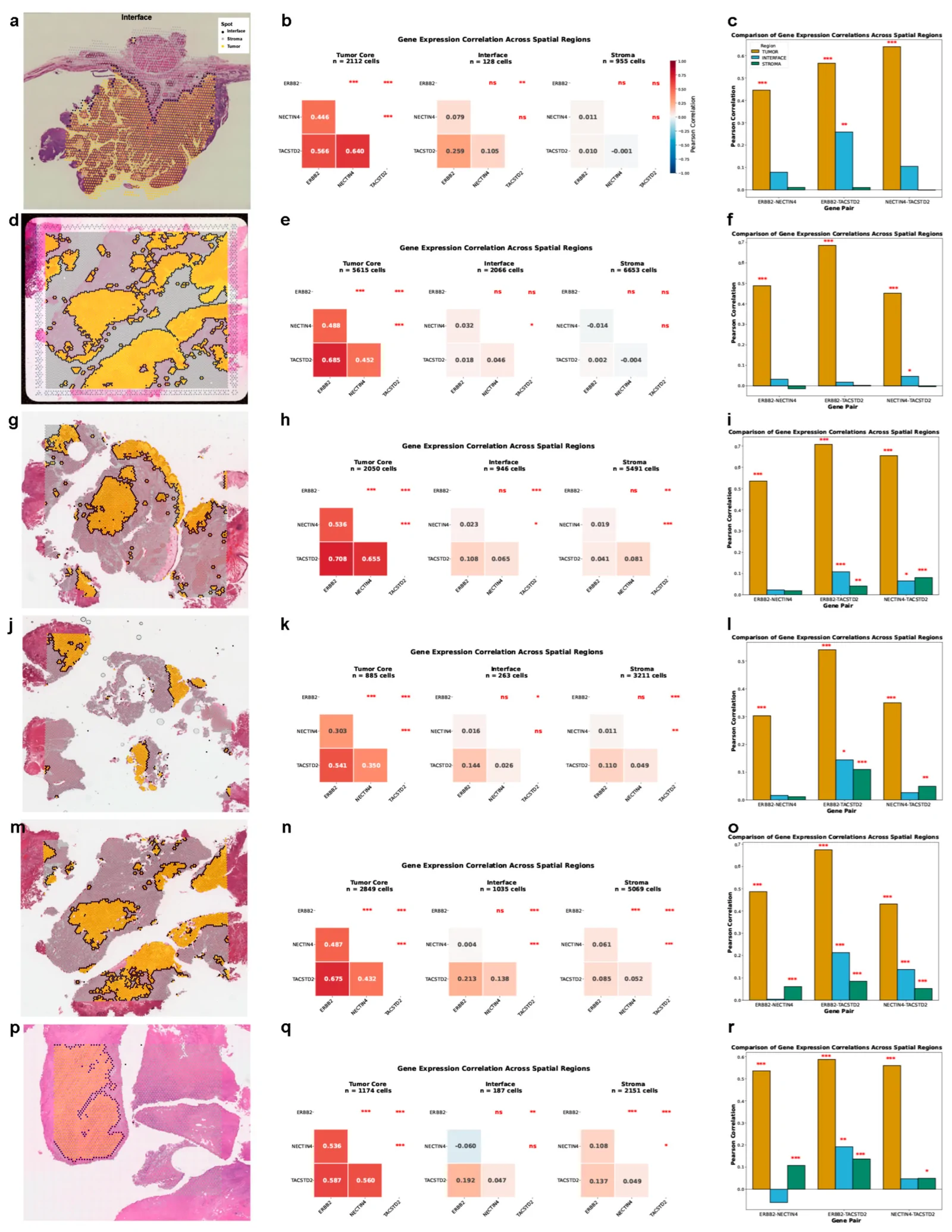

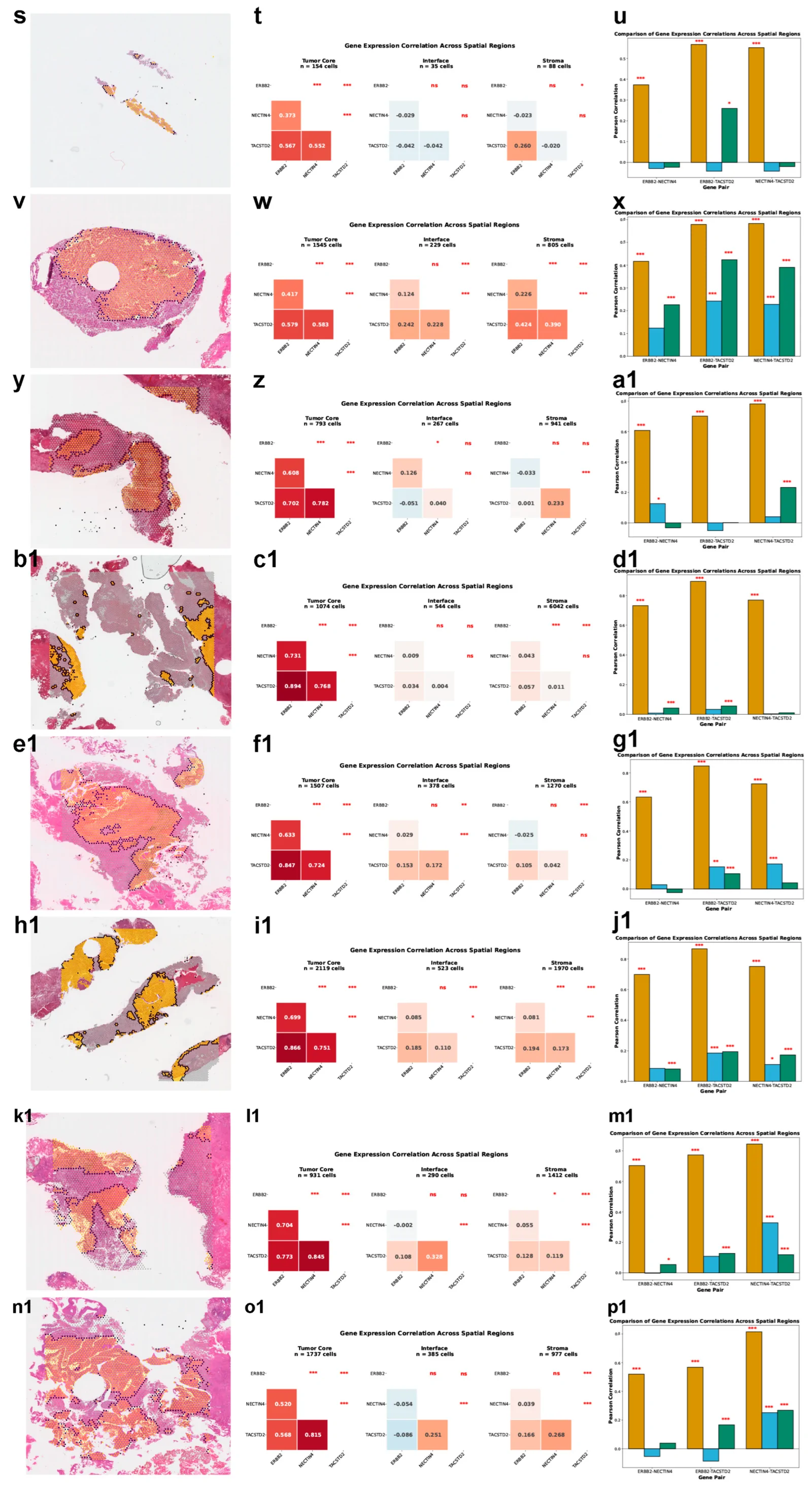
Correlations of *ERBB2*, *TACSTD2*, and *NECTIN4* expression across tumor core, interface, and stromal regions in multiple 10x Visium bladder cancer datasets: E-GEAD-1186 (sample 5: (**a**–**c**)); GSE246011 (sample B2: (**d**–**f**)); and GSE319536 (samples GSM9519394_BC01 (**g**–**i**), GSM9519397_BC04 (**j**–**l**), GSM9519400_BC07 (**m**–**o**), GSM9519401_BC08 (**p**–**r**), GSM9519402_BC09 (**s**–**u**), GSM9519403_BC10 (**v**–**x**), GSM9519405_BC12 (**y**–**a1**), GSM9519407_BC14 (**b1**–**d1**), GSM9519408_BC15 (**e1**–**g1**), GSM9519410_BC17 (**h1**–**j1**), GSM9519412_BC19 (**k1**–**m1**), and GSM9519414_BC21 (**n1**–**p1**)). For each sample, panels (**a**,**d**,**g**,**j**,**m**,**p**,**s**,**v**,**y**,**b1**,**e1**,**h1**,**k1**,**n1**) show spatial feature plots of gene expression distributions; panels (**b**,**e**,**h**,**k**,**n**,**q**,**t**,**w**,**z**,**c1**,**f1**,**i1**,**l1**,**o1**) display the corresponding correlation heatmaps; and panels (**c**,**f**,**i**,**l**,**o**,**r**,**u**,**x**,**a1**,**d1**,**g1**,**j1**,**m1**,**p1**) present bar plots of expression levels across the three tissue compartments. Two-sided *p*-values (Pearson correlation test) are denoted by asterisks: * *p* < 0.05, ** *p* < 0.01, and *** *p* < 0.001; ns, not significant.

To broaden our spatial analysis to early-stage disease, we examined the co-expression landscape of these three genes in non-muscle-invasive bladder cancer (NMIBC). We analyzed the 10x Visium spatial transcriptomic dataset E-GEAD-1186, containing seven NMIBC samples (samples 1–4, 6, and 7 in Figure 3; sample 5 in Figure 4a–c). Spot-level expression was overlaid onto matched histological sections—tumor core, interface, and stroma—and pairwise correlation analyses were conducted within each region.

Positive correlations were preserved across all seven NMIBC samples, aligning with the broader co-expression pattern documented throughout this study. Of the three gene pairs, *ERBB2* and *TACSTD2* displayed the strongest correlations within the tumor core compartment, with moderate-to-strong positive values—Pearson coefficients generally above 0.6 in the majority of specimens. Gene pairs involving *NECTIN4* were more variable yet remained predominantly positive. It is worth noting that correlation magnitudes across all pairs were generally attenuated in this NMIBC cohort relative to the GSE285715 dataset, hinting at possible divergence in co-expression strength between tumor subtypes or across independent study populations.

In several NMIBC specimens, correlation coefficients for particular gene pairs were relatively higher in the tumor core than in the stroma, with the interface zone showing low-to-intermediate values. Even so, the extent of regional fluctuation varied case by case, and no consistent spatial gradient emerged across the seven samples, suggesting that microenvironment-driven modulation of co-expression is subtle and specimen-dependent rather than systematic.

These observations extend our spatial findings from MIBC into the NMIBC setting and indicate that the concerted expression of *ERBB2*, *TACSTD2*, and *NECTIN4* is broadly conserved across different bladder cancer subtypes, albeit with appreciable variation in correlation strength between cohorts.

### 3.4. Validation of Spatial Co-Expression and High-Resolution Hotspot Mapping in Independent Bladder Cancer Visium Cohorts

**Figure 5:**
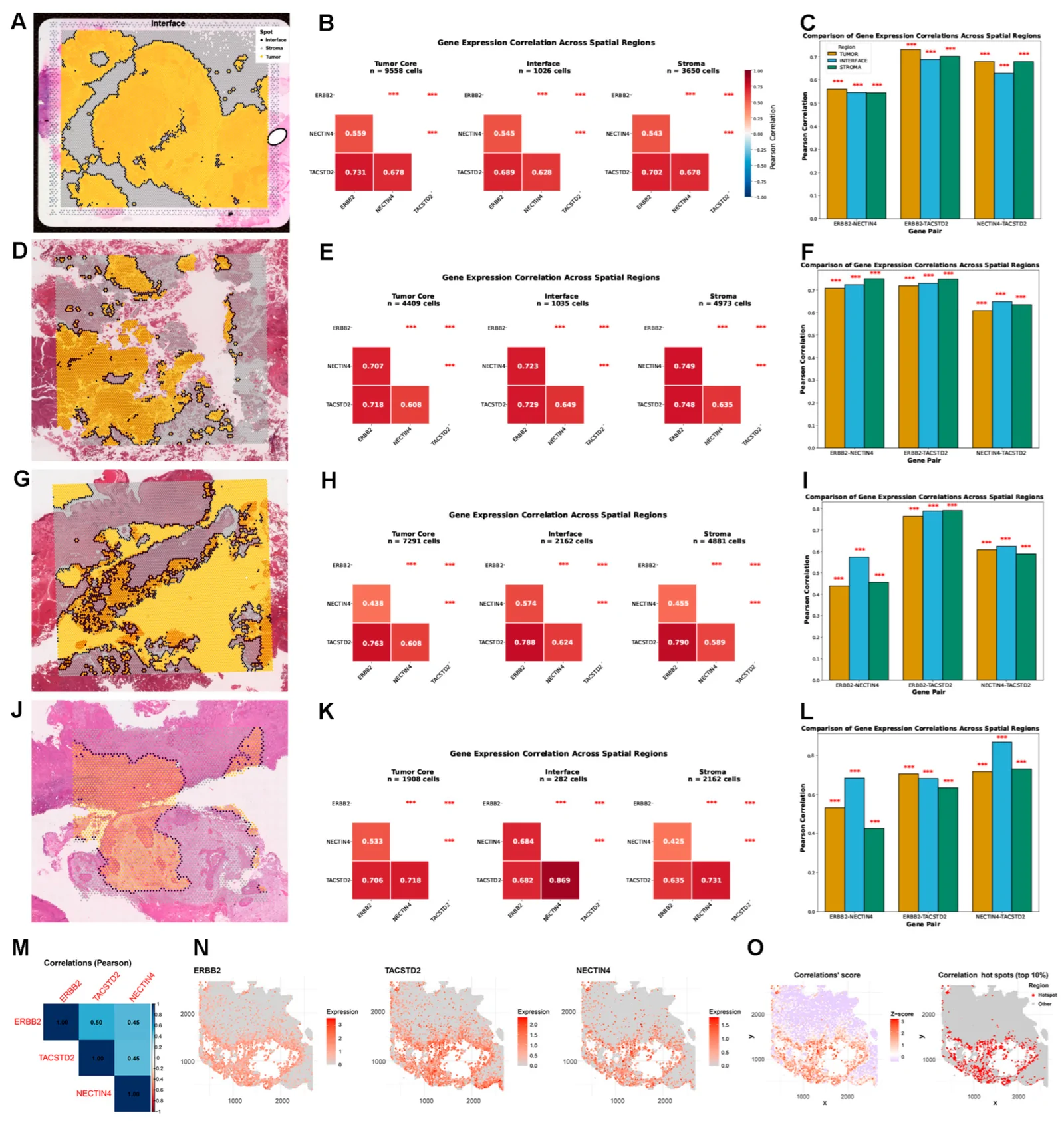
Correlations of *ERBB2*, *TACSTD2*, and *NECTIN4* expression across tumor core, interface, and stromal regions in the 10x Visium bladder cancer datasets GSE246011 (sample B1: (**A**–**C**)) and GSE319536 (sample GSM9519404_BC11: (**D**–**F**); sample GSM9519406_BC13: (**G**–**I**); sample GSM9519415_BC22: (**J**–**L**)), and co-expression of the three genes in the 10x Visium HD bladder cancer dataset GSE238145 (sample GSM8171243: (**M**–**O**)). For each Visium sample, panels (**A**,**D**,**G**,**J**) show spatial feature plots of gene expression distributions; panels (**B**,**E**,**H**,**K**) display the corresponding correlation heatmaps; and panels (**C**,**F**,**I**,**L**) present bar plots of expression levels across the three tissue compartments. For the Visium HD sample, panels (**M**,**N**,**O**) represent the correlation heatmap, spatial feature plots, and correlation scores with the top 10% of spatial correlation hotspots for the three genes, respectively. Two-sided *p*-values (Pearson correlation test) are denoted by asterisks: *** *p* < 0.001.

To further corroborate and broaden our spatial transcriptomic results in independent cohorts, we evaluated two samples from the 10x Visium dataset GSE246011 (sample B1 in Figure 5A–C; sample B2 in Figure 4d–f) and fifteen samples from GSE319536 (samples BC11, BC13, and BC22 in Figure 5D–L; samples BC01, BC04, BC07–BC10, BC12, BC14–BC15, BC17, BC19, and BC21 in Figure 4g–p1). Spot-level expression was mapped and pairwise correlations assessed in the tumor core, interface, and stromal compartments for each specimen. We additionally inspected the co-expression of the three genes in the 10x Visium HD dataset GSE238145 (sample GSM8171243), which delivers subspot-resolution spatial profiling to identify fine-scale co-expression domains defined as the uppermost 10% of spatial correlation hotspots (Figure 5M–O).

Across all four Visium samples presented in the main figure, positive pairwise correlations among *ERBB2*, *TACSTD2*, and *NECTIN4* were uniformly detected. The *ERBB2*–*TACSTD2* pair again stood out as the strongest correlation across tissue compartments in samples B1, BC11, and BC13, with Pearson coefficients typically near or above 0.7 in most specimens. Correlations entailing *NECTIN4* were more variable yet remained overwhelmingly positive. Regional comparisons revealed that the tumor core consistently yielded the highest coefficients for all three gene pairs. Among the full set of seventeen specimens, thirteen exhibited peak correlations for the *ERBB2*–*TACSTD2* pair (B1, BC11, BC13, B2, BC01, BC04, BC07–BC10, BC14, BC15, and BC17), while four showed the strongest correlations for *NECTIN4*–*TACSTD2* (BC22, BC12, BC19, and BC21). Cross-compartment comparison of correlation magnitudes—tumor core versus interface versus stroma—revealed no uniform spatial gradient, further supporting the notion that the overall co-expression framework is broadly upheld across spatial microenvironments despite modest local variation.

In the Visium HD dataset, which approaches single-cell spatial resolution, the co-expression patterns were further substantiated. Correlation heatmaps confirmed positive pairwise associations, and spatial feature plots demonstrated highly congruent expression distributions across *ERBB2*, *TACSTD2*, and *NECTIN4*. Spatial correlation analysis pinpointed the top decile of hotspots where coordinated expression of the three genes was most intense, uncovering discrete regional assemblies that may designate biologically meaningful niches for synchronous target expression. Interestingly, *ERBB2* and *TACSTD2* yielded the highest correlation coefficient (0.50) among the three gene pairs (Figure 5M).

Altogether, these results—spanning multiple independent spatial transcriptomic platforms and datasets, bolstered by the high-definition capacity of Visium HD—reinforce the dependability of *ERBB2*, *TACSTD2*, and *NECTIN4* co-expression in bladder cancer and advance our observations from conventional spatial transcriptomics to near-single-cell-resolution analysis.

### 3.5. mIF Profiling of HER2, Trop2, and Nectin-4 in Bladder Cancer and Paired Adjacent Normal Tissue

To evaluate the expression characteristics and tumor selectivity of three therapeutically actionable targets in bladder cancer, mIF staining for HER2 (magenta), Trop2 (cyan), and Nectin-4 (yellow) was performed on three paired sets of FFPE bladder cancer and matched adjacent normal tissues, with DAPI as a nuclear counterstain.

**Figure 6:**
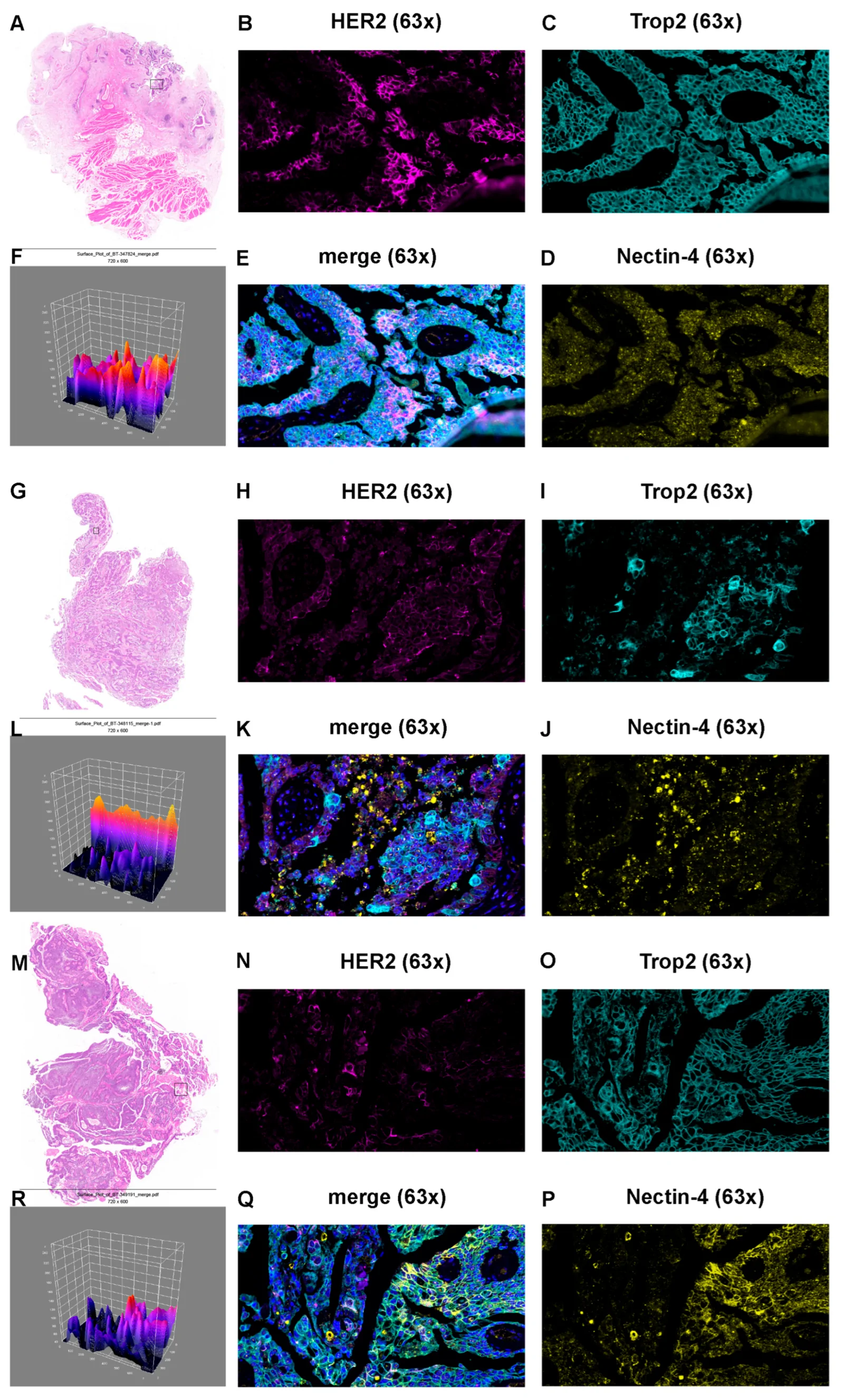
Hematoxylin and eosin (HE) staining and multicolor multiplex immunofluorescence (mIF) analysis of HER2, Trop2, and Nectin-4 expression in three bladder cancer tissues. (**A**,**G**,**M**) HE-stained sections of BT-347824 (sample 1), BT-348115 (sample 2), and BT-349191 (sample 3), respectively; black rectangles indicate the regions selected for mIF analysis. (**B**–**E**,**H**–**K**,**N**–**Q**) Corresponding mIF images at 63× magnification showing HER2 (magenta), Trop2 (cyan), and Nectin-4 (yellow) staining in individual channels, with merged images (**E**,**K**,**Q**) demonstrating colocalization. Nuclei were counterstained with DAPI (blue). Scale bar = 20 μm. (**F**,**L**,**R**) Three-dimensional surface plot reconstructions of the merged mIF images. The *Z*-axis represents cumulative fluorescence intensity (range, 40–240 arbitrary units), with peak elevations corresponding to areas of high marker co-expression and valleys indicating regions of low signal intensity. (mIF = multiplex immunofluorescence, HE = hematoxylin and eosin, DAPI = 4′,6-diamidino-2-phenylindole.)

Within tumor tissues, each of the three markers exhibited distinguishable staining profiles. Trop2 displayed the broadest and most homogeneous distribution across all three tumor specimens, with diffuse membranous and cytoplasmic signal covering the vast majority of neoplastic cells (≥90% of the tumor area). Nectin-4 showed moderate-to-extensive granular membranous/cytoplasmic distribution, with its spatial extent varying from focal regions in sample 1 (BT-347824) to more widespread zones in samples 2 (BT-348115) and 3 (BT-349191). HER2 showed the greatest heterogeneity, with membranous positivity confined to scattered tumor cell clusters in sample 1 (estimated at 10–20% positivity), patchy, moderate expression in sample 2 (20–25%), and broader, sheet-like membranous positivity in sample 3 (40–50%). Notably, co-expression assessment revealed extensive spatial overlap between Trop2 and Nectin-4 in all three tumors, whereas HER2-positive cell populations intersected only partially with Trop2/Nectin-4-positive territories, implicating HER2 as a marker of a distinct molecular subset within these tumors. Triple co-expression (HER2+/Trop2+/Nectin-4+) was detected in limited tumor cell subpopulations, reaching its greatest extent in sample 3 (Figure 6A–E,G–K,M–Q).

**Figure 7:**
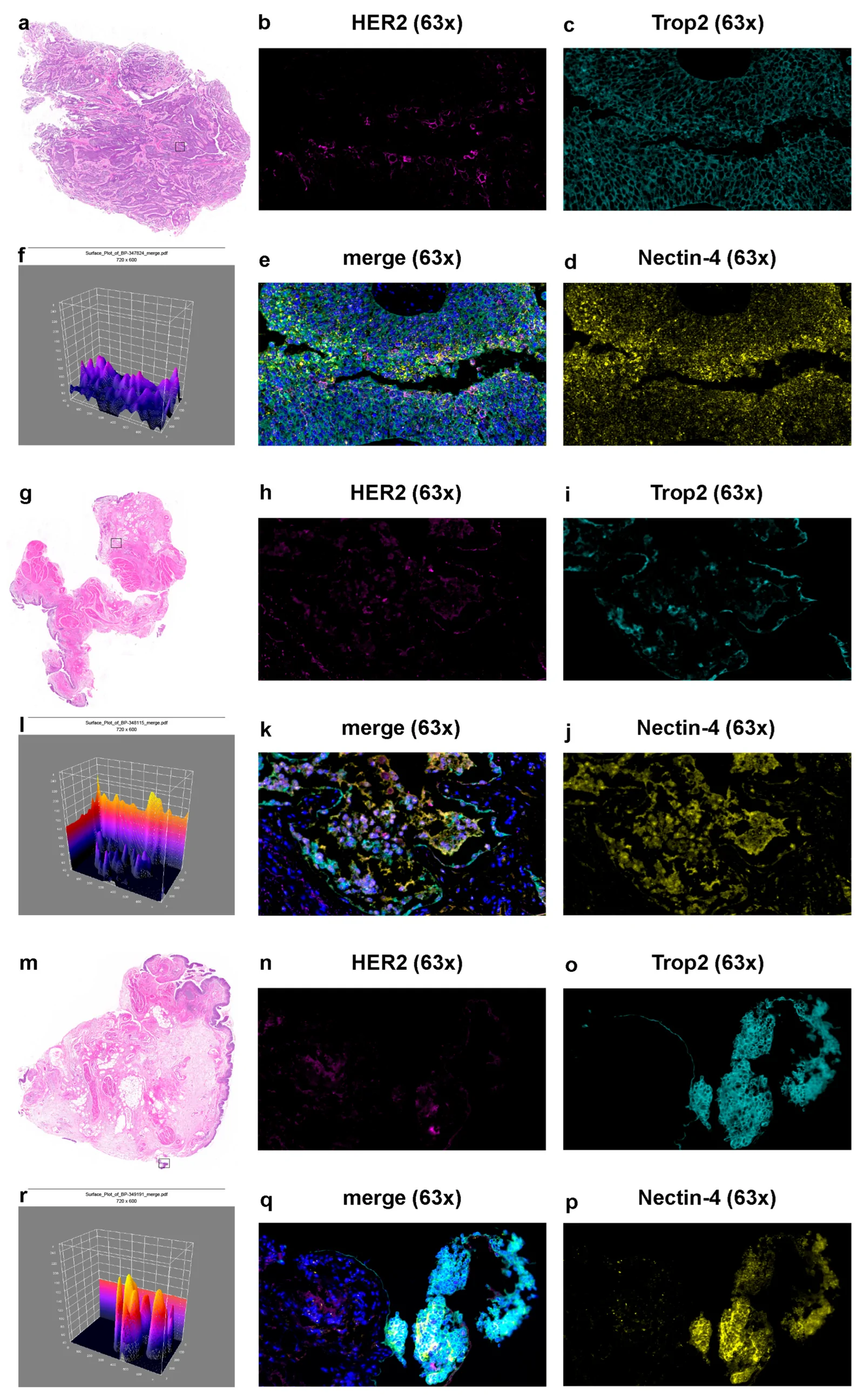
HE staining and multicolor mIF analysis of HER2, Trop2, and Nectin-4 expression in paired adjacent normal tissues. (**a**,**g**,**m**) HE-stained sections of BP-347824 (normal counterpart of sample 1), BP-348115 (normal counterpart of sample 2), and BP-349191 (normal counterpart of sample 3), respectively; black rectangles indicate the regions selected for mIF analysis. (**b**–**e**,**h**–**k**,**n**–**q**) Corresponding mIF images at 63× magnification showing HER2 (magenta), Trop2 (cyan), and Nectin-4 (yellow) staining in individual channels, with merged images (**e**,**k**,**q**) demonstrating colocalization. Nuclei were counterstained with DAPI (blue). Scale bar = 20 μm. (**f**,**l**,**r**) Three-dimensional surface plot reconstructions of the merged mIF images. The *Z*-axis represents cumulative fluorescence intensity (range, 40–240 arbitrary units). In contrast to the prominent signal peaks observed in the corresponding tumor tissues (Figure 6), the adjacent normal tissues exhibited a markedly flattened topographical profile, reflecting the near-absence of HER2, Trop2, and Nectin-4 expression. (mIF = multiplex immunofluorescence, HE = hematoxylin and eosin, DAPI = 4′,6-diamidino-2-phenylindole.)

In pronounced contrast, matched adjacent normal tissues showed dramatically attenuated signals for all three markers. The Trop2 expression was severely curtailed, limited to weak positivity in residual epithelial remnants or glandular architecture. Nectin-4 staining appeared sparse and punctate, largely absent from the normal parenchyma. HER2 signal was virtually undetectable, with only faint, diffuse background fluorescence. Triple co-expression was essentially non-existent across all adjacent normal specimens (Figure 7a–e,g–k,m–q).

Surface plots comparing tumor samples (BT-347824, BT-348115, BT-349191) with their paired adjacent normal counterparts (BP-347824, BP-348115, BP-349191) used the *Z*-axis to represent cumulative fluorescence intensity (range 40–240 arbitrary units). Within tumor specimens, prominent signal peaks with maximum Z-values nearing 240 emerged predominantly over tumor cell nests, demarcating areas of robust Trop2, Nectin-4, and HER2 co-expression. Sample 3 (BT-349191) displayed the most elevated and extensive peak architecture among the three tumors, consistent with its higher overall expression. In contrast, surface plots of paired adjacent normal tissues (BP series) produced a markedly flattened landscape, with Z-values generally remaining below 100–120 arbitrary units and devoid of high-elevation peaks. Occasional low-amplitude undulations corresponded to residual epithelial structures, but the overall topographic profile remained conspicuously subdued relative to the neoplastic counterparts (Figure 6F,L,R and Figure 7f,l,r).

Taken together, these findings document a pronounced tumor-specific upregulation of Trop2, Nectin-4, and HER2 in bladder cancer relative to paired adjacent normal tissue. The divergent expression profiles across the three tumors further underscore the molecular heterogeneity of bladder cancer, where Trop2 and Nectin-4 represent broadly applicable ADC targets, whereas HER2 shows variable expression that may guide patient selection for anti-HER2-directed agents. The near-complete absence of these markers in adjacent normal tissue argues for an acceptable therapeutic index for ADC-based approaches. Three-dimensional surface plot analysis offered an intuitive topographic representation of the differential signal intensity landscapes between neoplastic and normal tissue, serving as a quantitative complement to the qualitative mIF observations. However, the mIF data from three paired specimens provide preliminary protein-level evidence consistent with the transcriptomic co-expression patterns, although the limited sample size precludes definitive conclusions.

## 4. Discussion

This study presents a comprehensive, multi-resolution characterization of the expression relationships among three clinically pertinent ADC targets—*ERBB2* (HER2), *TACSTD2* (Trop2), and *NECTIN4* (Nectin-4)—in bladder cancer, combining bulk transcriptomics, spatial transcriptomics, and mIF across 1628 bulk samples and 31 spatial specimens. Our data demonstrate a consistent, predominantly positive co-expression framework connecting these three genes that persists across datasets, disease subtypes, and histological compartments and is further supported by preliminary protein-level evidence from mIF.

In ten independent bulk RNA-seq cohorts encompassing both MIBC and NMIBC cases, we documented moderate-to-strong positive correlations among all three genes. The tightest association was between *TACSTD2* and *NECTIN4*, with coefficients reaching 0.969, pointing to especially robust co-regulation of this pair. Correlations between *ERBB2* and the other two genes fluctuated more across datasets but stayed uniformly positive. The complete absence of inverse correlations across all cohorts supports a model wherein these genes are not reciprocally antagonized but instead engage in a shared expression program, possibly governed by overlapping transcriptional regulatory networks or convergent upstream signaling cascades. This hypothesis aligns well with our previously reported findings that a basal-like malignant epithelial cell is regulated by *RUNX3*, architecting an immunosuppressive microenvironment in bladder cancer [26]. These results establish a transcriptomic platform for the synchronous expression of these targets, with consequences for treatment strategies that aim to engage several ADC antigens at once.

This co-expression pattern is further corroborated by a large-scale genomic profiling study by Bilen et al., which analyzed 4743 urothelial carcinoma tumors and demonstrated that *ERBB2*-high tumors exhibited significantly higher expression of *NECTIN4* (12 vs. 8 median TPM, *p* < 0.001) and *TACSTD2* (366 vs. 74 median TPM, *p* < 0.001) compared to *ERBB2*-low tumors. Notably, they also reported a high concordance between HER2 immunohistochemistry (IHC) and *ERBB2* RNA expression (79% of *ERBB2*-high tumors were HER2-positive by IHC), supporting the clinical translatability of transcript-level assessments of these targets. Their finding that *ERBB2*-high tumors had a higher prevalence of pathogenic mutations in *pTERT*, *ERBB2*, and *ELF3*, alongside elevated expression of *NECTIN4* and *TACSTD2*, suggests that these three ADC targets may be part of a coordinated transcriptional program linked to specific genomic backgrounds in urothelial carcinoma. Moreover, the observed better overall survival in *ERBB2*-high tumors (HR 1.34, *p* < 0.001), while seemingly counterintuitive given the association of HER2 with aggressive disease in other cancer types, may reflect the more luminal-like molecular subtype frequently associated with *ERBB2* overexpression, which carries a more favorable prognosis relative to basal/squamous subtypes [27]. This survival advantage adds a layer of complexity to the clinical interpretation of *ERBB2* co-expression with *TACSTD2* and *NECTIN4* and warrants further investigation in prospective cohorts.

A persistent concern in bulk transcriptomic analyses is that apparent correlations may reflect artifacts of sample pooling that obscure genuine intratumoral spatial heterogeneity. Our spatial transcriptomic evaluation across multiple 10x Visium collections—spanning primary tumors, adjacent normal tissue, and NMIBC specimens—directly tackles this issue. Positive correlations among *ERBB2*, *TACSTD2*, and *NECTIN4* were faithfully reproduced at the spot level throughout the tumor core, the tumor–stroma interface, and the stromal compartment. While subtle variations in correlation strength were sometimes noted between compartments—the tumor core occasionally showing the top coefficients—no consistent directional spatial gradient materialized. This implies that the co-expression relationships are fundamentally robust across tissue niches rather than being dominated by a single compartment. These findings receive additional support from the Visium HD dataset, which, at near-single-cell resolution, corroborated positive pairwise correlations and permitted the delineation of discrete spatial hotspots representing the top decile of co-expression—biological microenvironments where synchronous target expression is most concentrated.

The retention of co-expression across tumor subtypes also deserves attention. Dernbach et al. [28] further demonstrated that HER2 expression was associated with urothelial-like and genomically unstable molecular subtypes, whereas Trop2 was widely expressed except in the mesenchymal-like subtype, and Nectin-4 showed an absence of staining in basal, mesenchymal-like, and small-cell/neuroendocrine-like subtypes. These subtype-specific expression patterns may underlie the variable correlation magnitudes we observed across cohorts and highlight the importance of molecular subtyping in interpreting target co-expression. While correlation magnitudes were generally lower in the NMIBC cohort (E-GEAD-1186) than in GSE285715, and the MIBC cohort GSE319536 showed variable correlation strengths, the positive directionality was uniformly preserved. This indicates that the co-expression program is broadly maintained through different disease stages, though its intensity may fluctuate with tumor biology, grade, or stage—a premise that merits further testing in larger, stage-stratified series.

The mIF analyses provided complementary—albeit preliminary—protein-level evidence and additional biological insight into the expression and spatial distribution of the three markers. All three protein markers—HER2, Trop2, and Nectin-4—were detectable in bladder tumor tissue with distinct patterns and intensities. Trop2 exhibited the most uniform and expansive coverage (~90% of tumor area), aligning with its reputation as a pan-carcinoma antigen and buttressing its broad applicability as an ADC target in urothelial cancers. This is consistent with findings in upper tract urothelial carcinoma (UTUC), where Shi et al. [29] demonstrated that Trop-2 expression remained consistently high across tumor grades and stages. Nectin-4 displayed moderate-to-extensive membranous/cytoplasmic distribution across samples. Shi et al. similarly reported that Nectin-4 expression in UTUC was significantly elevated in high-grade invasive tumors compared to low-grade tumors and showed marked increases with pathological stage progression. Moreover, they identified a significant positive correlation between Nectin-4 expression and the Ki-67 proliferation index (r = 0.4826, *p* < 0.001), whereas the correlation between Trop-2 and Ki-67 was weak and non-significant. These findings suggest that Nectin-4 may be functionally linked to tumor proliferation, potentially distinguishing its biological role from that of Trop-2 despite their extensive co-expression. HER2 was the most heterogeneous target in our analysis, with positivity spanning from 10–20% to 40–50% across specimens, highlighting the well-recognized intratumoral variability of this target and reinforcing the importance of rigorous patient selection for anti-HER2 ADC therapy. The clinical significance of HER2 heterogeneity has been further amplified by the recent tumor-agnostic approval of trastuzumab deruxtecan (T-DXd) for HER2-overexpressing (IHC 3+) solid tumors, as reviewed by Ko et al. [30]. The DESTINY-PanTumor02 trial [8] demonstrated that T-DXd achieved an objective response rate of 37.1% across all HER2-expressing solid tumors and 61.3% in IHC 3+ tumors, leading to the first tumor-agnostic approval for an ADC. This evolving landscape underscores the need for accurate and reproducible HER2 assessment, where AI-assisted IHC interpretation and NGS-based detection of *ERBB2* copy number gain have emerged as complementary strategies to address interobserver variability.

Co-expression dissection of the mIF data uncovered extensive Trop2–Nectin-4 overlap across all three tumors, whereas HER2-positive cell populations intersected only partially with Trop2/Nectin-4-positive regions, suggesting that HER2 may delineate a molecularly distinct compartment within the broader Trop2/Nectin-4-expressing tumor landscape. Triple-positive subpopulations were identifiable but remained circumscribed, attaining their highest representation in the sample with the greatest HER2 coverage. Shi et al. reported that in UTUC, a subset of patients (25%) exhibited high expression of Nectin-4 in tumor tissue with low expression in paired benign tissue, and a similar pattern was observed for Trop-2, corroborating the tumor-specific upregulation we observed at the protein level. These convergent findings across lower and upper tract urothelial carcinomas suggest that the tumor selectivity of these targets is a conserved feature of urothelial malignancies.

From a biological perspective, these observations generate the hypothesis that patients whose tumors exhibit extensive Trop2 and Nectin-4 co-expression might derive comparable benefit from either anti-Trop2 or anti-Nectin-4 ADC therapy, or potentially from sequential or combined regimens, while the presence of discrete HER2-positive subpopulations might justify incorporating an anti-HER2 agent. The constrained triple-positive compartment further suggests that multi-target ADC strategies could engage a larger aggregate fraction of tumor cells. However, clinical validation of this hypothesis—and any determination of whether co-expression patterns predict differential responses to single-agent versus sequential versus combination regimens—awaits dedicated prospective studies.

An important distinction must be drawn between the biological observations reported here and their potential therapeutic implications. Our study demonstrates that *ERBB2*, *TACSTD2*, and *NECTIN4* transcripts and proteins exhibit positively correlated expression across multiple scales of analysis in bladder cancer. This correlative finding supports the biological plausibility that these targets are part of a coordinated expression program. However, whether this co-expression translates into differential clinical benefit from single-agent, sequential, or combination ADC regimens remains entirely unknown and cannot be inferred from expression data alone. Prospective clinical trials with embedded biomarker programs—in which pre-treatment tumor specimens are profiled for these three targets and outcomes are compared across expression-defined subgroups—are required to establish the clinical utility of co-expression patterns for therapeutic decision-making. Until such evidence is available, discussion of sequential or combination ADC strategies based on co-expression data should be regarded as hypothesis generation only.

Arguably, the most clinically consequential observation from the mIF analysis is the pronounced tumor-selective upregulation of all three markers. Matched adjacent normal tissues showed markedly diminished or virtually absent signals for HER2, Trop2, and Nectin-4, with triple co-expression essentially absent in normal specimens. The three-dimensional surface plot reconstructions delivered an intuitive topographic visualization of this disparity: tumor tissues produced prominent signal peaks with maximum cumulative fluorescence intensities approaching 240 arbitrary units, while adjacent normal tissues yielded a flattened topography with intensities typically below 100–120 units. This preferential expression pattern supports a favorable therapeutic window for ADC strategies directed at these three antigens. In UTUC, Shi et al. similarly found that in 24 paired samples, tumor tissue expressed significantly higher levels of Nectin-4 (*p* < 0.05) and Trop-2 (*p* < 0.05) compared to non-tumor urothelial tissue, reinforcing the generalizability of this observation across the entire urothelial tract. The near-complete absence of these molecules in normal bladder epithelium suggests that on-target, off-tumor toxicity may be clinically manageable, consistent with the safety experience of EV, SG, and anti-HER2 ADCs in urothelial carcinoma.

The spatial heterogeneity of these targets also carries implications for clinical biopsy strategies. Dernbach et al. demonstrated that a single biopsy may be insufficient for reliable assessment of HER2 and Nectin-4 expression, with four biopsies needed for accurate evaluation of these two markers. Our spatial transcriptomic data, while not directly addressing biopsy adequacy, corroborate the existence of regional variation in expression and co-expression intensity. These findings collectively argue for multi-region sampling in clinical trials and practice when these ADC targets are used for patient stratification.

Several constraints of this work warrant acknowledgment. First, although the study encompasses 1628 bulk samples and 31 spatial specimens, protein-level assessment is limited to three paired tumor–normal specimens. This sample size is insufficient to support broad biological or clinical generalization; the protein-level findings should therefore be regarded as preliminary and illustrative. Larger multi-institutional cohorts with standardized mIF and immunohistochemistry protocols are needed to verify the broader applicability and clinical significance of these observations. Second, the spatial transcriptomic platforms employed (10x Visium and Visium HD) measure mRNA rather than protein; while our mIF data supply complementary protein-level corroboration, the relationship between transcript abundance and protein abundance for these targets in defined spatial contexts requires further exploration. Third, this study is descriptive and does not elucidate the causal underpinnings of the observed co-expression. The transcriptional or epigenetic machinery responsible for the coordinated expression of *ERBB2*, *TACSTD2*, and *NECTIN4* remains to be defined. Fourth, the therapeutic implications of co-expression—such as whether dual-positive tumors derive incremental clinical benefit from combination ADC regimens—lie beyond the reach of our correlative dataset and must be addressed in prospective clinical trials. Fifth, the bulk RNA-seq cohorts were heterogeneous with respect to disease stage, treatment history, and specimen origin. While this heterogeneity strengthens the generalizability of consistent co-expression patterns observed across datasets, it also introduces potential confounders that cannot be fully controlled. In particular, prior systemic therapy—as in the IMvigor210 cohort (all post-platinum)—could influence target gene expression. We note, however, that the co-expression patterns in IMvigor210 were generally consistent with those in treatment-naive cohorts, suggesting that prior platinum exposure does not fundamentally disrupt the observed correlations. Nevertheless, the impact of prior therapy on ADC-target co-expression warrants dedicated prospective investigation.

## 5. Conclusions

In summary, we demonstrate that *ERBB2*, *TACSTD2*, and *NECTIN4* are consistently co-expressed in bladder cancer across bulk transcriptomic and spatial transcriptomic levels, with supporting protein-level evidence from three paired tumor–normal specimens. This co-expression pattern endures across tumor subtypes, spatial compartments, and individual specimens, with pronounced tumor selectivity relative to adjacent normal tissue. These observations provide a descriptive, multi-resolution characterization of the co-expression landscape of these three ADC targets. The marked tumor-selective upregulation of these targets is consistent with a favorable therapeutic index, and the identification of distinct co-expression patterns—notably the extensive Trop2/Nectin-4 overlap alongside more restricted HER2 positivity—may generate testable hypotheses for biomarker-informed patient stratification in future clinical studies. We emphasize that the clinical utility of these co-expression patterns for guiding ADC-based therapeutic strategies remains to be established in prospective trials with adequately powered validation cohorts.

## Figures and Tables

**Table 1:** Cohort summary for this research.

Dataset	Platform	Sample Size (*n*)	Disease Stage	Sample Origin	Treatment Status at Collection	Key Clinical Characteristics
TCGA-BLCA	Bulk RNA-seq	408	MIBC (100%)	Primary tumor	Treatment-naive (majority)	Staging, grade, molecular subtypes available
IMvigor210	Bulk RNA-seq	195	Metastatic UC (100%)	Primary tumor and metastatic lesions	Post-platinum (all patients)	Anti-PD-L1 treatment outcomes available
E-MTAB-1803	Bulk RNA-seq	73	MIBC (100%)	Primary tumor	Treatment-naive	Matched normal available
GSE31684	Bulk RNA-seq	93	MIBC (84%)	Primary tumor	Treatment-naive	Grade, survival data available
GSE133624	Bulk RNA-seq	65 (36 tumor + 29 normal)	-	Primary tumor and adjacent normal	Treatment-naive	Matched normal available (22 pairs)
GSE216037	Bulk RNA-seq	52	-	-	-	TP53 mutation-related study
GSE236932	Bulk RNA-seq	63 (38 tumor + 25 normal)	MIBC + NMIBC	Primary tumor and peritumoral tissues	Treatment-naive	Whole-transcriptome sequencing
GSE248167	Bulk RNA-seq	51	-	-	-	Molecular subtyping study
GSE279390	Bulk RNA-seq	562	MIBC	-	Post-neoadjuvant chemotherapy	VESPER trial samples; chemotherapy response data available
GSE328930	Bulk RNA-seq	66	-	-	-	-
GSE285715	10x Visium	6 sections (3 tumor + 3 normal)	-	Primary tumor + adjacent normal	Treatment-naive	-
E-GEAD-1186	10x Visium	7 sections	NMIBC	Primary tumor	Treatment-naive	Staging, grade, molecular subtypes available
GSE246011	10x Visium	2 sections	MIBC	Primary tumor	Treatment-naive	-
GSE319536	10x Visium	22 sections	MIBC	Primary tumor	Treatment-naive	Staging available
GSE238145	10x Visium HD	10 sections	Invasive bladder carcinoma	Primary tumor	Treatment-naive	-

**Table 2:** Demographic and clinicopathological features of the three bladder cancer samples and three paired adjacent normal samples used for multiplex immunofluorescence staining.

Sample	Number	Gender	Age	Pathology	Stage	Grade	Invasive	Tumor Thrombus in Blood Vessels	Nerve Invasion
Bladder cancer 1	BT-347824	Mmale	57	Urothelial carcinoma	T4aN1M0	High	Invasive	yes	yes
paired adjacent normal 1	BP-347824	Male	57						
Bladder cancer 2	BT-348115	Male	69	Urothelial carcinoma	T1N0M0	High	Invasive	no	no
paired adjacent normal 2	BP-348115	Male	69						
Bladder cancer 3	BT-349191	Female	68	Mixed: urothelial carcinoma; adenocarcinoma; plasmacytoid; transparent cell	T2N0M0	High	Invasive	yes	no
Paired adjacent normal 3	BP-349191	Female	68						

## Data Availability

All datasets analyzed in this study are publicly available, and no new sequencing or proteomic data were created in this study. The code used for data processing, correlation analyses, and visualization in this study is available on GitHub at https://github.com/sanheliu/ADC-Targets-ERBB2-TACSTD2-NECTIN4-in-Bladder-Cancer-Multi-Scale-Landscape (accessed on 17 July 2026). The repository includes Python scripts for bulk RNA-seq correlation analysis, spatial transcriptomic data processing with scanpy, and R scripts for Visium HD analyses. All analyses were performed using open-source tools and publicly available datasets as described in Section 2.
